# Emerging hybridity: comparing UK healthcare regulatory arrangements

**DOI:** 10.1108/JHOM-06-2016-0109

**Published:** 2017-06-19

**Authors:** Joy Furnival, Kieran Walshe, Ruth Boaden

**Affiliations:** 1 Health Management Group, Alliance Manchester Business School, University of Manchester, Manchester, UK; 2 Collaboration for Leadership in Applied Health Research and Care Greater Manchester, Alliance Manchester Business School, University of Manchester, Manchester, UK

**Keywords:** Quality Improvement, Quality, Regulation, Compliance, Quality assurance, Hybridity

## Abstract

**Purpose:**

Healthcare regulation is one means to address quality challenges in healthcare systems and is carried out using compliance, deterrence and/or improvement approaches. The four countries of the UK provide an opportunity to explore and compare different regulatory architecture and models. The purpose of this paper is to understand emerging regulatory models and associated tensions.

**Design/methodology/approach:**

This paper uses qualitative methods to compare the regulatory architecture and models. Data were collected from documents, including board papers, inspection guidelines and from 48 interviewees representing a cross-section of roles from six organisational regulatory agencies. The data were analysed thematically using an a priori coding framework developed from the literature.

**Findings:**

The findings show that regulatory agencies in the four countries of the UK have different approaches and methods of delivering their missions. This study finds that new hybrid regulatory models are developing which use improvement support interventions in parallel with deterrence and compliance approaches. The analysis highlights that effective regulatory oversight of quality is contingent on the ability of regulatory agencies to balance their requirements to assure and improve care. Nevertheless, they face common tensions in sustaining the balance in their requirements connected to their roles, relationships and resources.

**Originality/value:**

The paper shows through its comparison of UK regulatory agencies that the development and implementation of hybrid models is complex. The paper contributes to research by identifying three tensions related to hybrid regulatory models; roles, resources and relationships which need to be managed to sustain hybrid regulatory models.

## 1. Introduction

In the four countries of the UK different healthcare regulatory arrangements have developed, which provides an opportunity to study emerging hybrid healthcare regulatory models.

The purpose of this paper is:

- to understand and analyse healthcare regulatory models within the UK;
- to identify regulatory model developments; and
- to understand the tensions related to the development of hybrid regulatory models.

The paper is organised as follows: first, the background outlines relevant regulatory theoretical concepts. Second, the method and scope of the paper is detailed. Third, the current regulatory architecture and models are detailed. This outlines an emerging trend towards the use of hybrid regulatory models. Three tensions identified from the use of hybrid regulatory models are described in the findings and discussion. The paper concludes by outlining the contribution of the work.

## 2. Healthcare regulation

Regulation can be defined as “sustained and focussed control exercised by a public agency over activities which are valued by a community” (Selznick, 1985, p. 363). Regulation arises for several reasons, including the need to adjust for market failures, unequal bargaining power, critical goods shortages or moral hazards (Feintuck, 2012), where the consumer pays indirectly for services or to reduce discrimination and further social solidarity (Prosser, 2006). Healthcare regulation addresses stakeholders’ demands for improved performance.

Walshe (2003b) describes three main aims of regulation: improvement, assurance and accountability together with three regulatory models: compliance, deterrence (Reiss, 1984) and responsive (Ayres and Braithwaite, 1992). Deterrence models assume that organisations are “amoral” (Bardach and Kagan, 1982) and will deliberately break rules, thus compliance must be enforced. In contrast, compliance models assume organisations will seek to comply with regulatory requirements if they can, and focus on persuasion and encouragement rather than formal or punitive enforcement.

Responsive regulation emphasises the combination of both “deterrence” and “compliance” models (Ayres and Braithwaite, 1992; Braithwaite, 2011). This flexible model allows regulatory agencies to choose their approach depending on performance or risk levels (Parker, 2013). Regulatory intervention escalates (or de-escalates) through a hierarchy as performance changes. This form of regulation assumes that trust-based models will improve care more effectively (Ayres and Braithwaite, 1992; Braithwaite, 2011). It is more suited to organisations and sectors seeking long-term improvement but it is challenging to sustain with large numbers of organisations.

In this paper, a responsive regulatory model is described as a “hybrid” model, and the term is used to refer to a regulatory agency that uses a combination of deterrence and compliance models.

McDermott *et al.* (2015) describe hybrid regulatory agencies who simultaneously use compliance and deterrence models to support performance improvement, “hybridity” is a concept widely used to describe organisational responses to changes in governance (Skelcher and Smith, 2015) and it is argued that it may support the reconfiguration of organisational models as circumstances change, accommodating multiple demands and developing new ideas (Miller *et al.*, 2008; Borys and Jemison, 1989). However, hybridity may also lead to the disruption of existing professional communities and identities (Smith, 2014) and unstable organisations which may fracture under sustained pressure (Denis *et al.*, 2015). Fischer and Ferlie (2013) argue that regulatory regimes consist of various values, norms and instruments that cannot be readily combined. It is sometimes argued that structural separation may be needed to manage the tensions that arise (Kippist and Fitzgerald, 2009; McDermott *et al.*, 2015). Nevertheless, hybrid models can produce stable states and can improve performance relative to traditional models (Miller *et al.*, 2008). This paper suggests that hybrid regulatory models may be more effective in producing improvement, but are more complex to design and implement and difficult to sustain.

There are three main regulatory processes: direction, detection and enforcement. Direction defines standards and removes systemic barriers through the provision of external policy impetus. Detection refers to the measurement and monitoring of performance. Enforcement is central to regulation and covers the methods used to educate, persuade, influence and force behavioural change (Hutter, 1989; Walshe and Shortell, 2004).

Regulation provides valuable feedback supporting improvement and requires high standards of performance to be maintained which otherwise they may not be (Gunningham, 2012). Despite this, it is often critiqued. Flodgren *et al.* (2011) finds a lack of effectiveness, other problems include high costs (Ng, 2013), inflexibility (Brennan, 1998), tunnel vision, (Mannion *et al.*, 2005), inhibiting innovation (Stewart, 1981), provider capture (Boyd and Walshe, 2007), ritualistic and bureaucratic compliance (Braithwaite *et al.*, 2007), a short term focus (Walshe, 2003b), loss of autonomy (Donabedian, 1988) and generating fear (Berwick, 2013).

Recognising the limits of deterrence and compliance regulatory models, alternative supportive and more contingent models using professionalism and improvement support are increasingly proposed (Ham, 2014). These models are intended to ensure healthcare systems can deliver high performance and can be viewed as a variation or development of responsive regulation. However, there are few studies (e.g., McDermott *et al.*, 2015) analysing the impact and influence of these emerging models. This paper contributes through comparative analysis of healthcare regulatory agencies across the UK.

## 3. Methodology

This paper focusses on the six UK organisational regulatory agencies: Care Quality Commission (CQC), Monitor, Trust Development Authority (TDA) in England[1] and Healthcare Inspectorate Wales (HIW), Healthcare Improvement Scotland (HIS) and the Regulatory and Quality Improvement Authority (RQIA) in Northern Ireland. Hospital-based care is the main area of focus for this paper, since it accounts for the majority of healthcare expenditure in the UK and all four countries oversee this healthcare area.

The study identified and analysed healthcare policy documents from each devolved country that included information related to regulatory purpose, strategy and results. Following permission to proceed after ethical review, the directors of policy or regulation within each regulatory agency were contacted to discuss organisational participation within the study. All six agencies agreed, and a cross-section of employees was interviewed. The interviewees held roles including board-level executives and inspectors, with a mixture of clinical and non-clinical backgrounds from each regulatory agency. Participation was voluntary and confidential. The interviews took place between October 2014 and April 2015.

The study used a semi-structured interview process based on the documentary analysis (Thomas, 1993). Questions included “what is the aim and purpose of this agency?”, and “what types of interventions do you use and why?” Testing of the questions took place through five pilot interviews. Interviewees were provided with copies of the transcripts to allow for any clarifications. The use of interviews allowed complex, subjective and sometimes contradictory data to be collected from participants that could not be gathered from other approaches. The data collected from interviews and documents were analysed iteratively on NVivo data analysis software, using an a priori coding template developed from the literature. This was used to compare the current regulatory architectures, models and aims and supported the organisation and interpretation of data through the identification of themes.

## 4. Results

The results are presented in two sections; the first section introduces the landscape of regulatory architecture in the UK. An overview of the six regulatory agencies is provided in Table I. This architecture comparison is used to identify the regulatory models in use and development, showing that hybrid regulatory models are emerging. The second section details three tensions arising from the emergence of hybrid regulatory models.

### The UK regulatory architecture

HIS was established in 2011. It combines a number of predecessor Scottish organisations. Its aim and purpose is to advance improvement in healthcare in Scotland, and to support providers to deliver safer, more effective and more person-centred care. HIS does not review social care services; a separate inspectorate oversees this.

HIW was established in 2004 and it is a unit of the Welsh Assembly Government. It has wide-ranging responsibilities including inspection of health boards and trusts, the regulation of independent healthcare providers, general practices, pharmacies and dental practices. Like HIS, HIW does not oversee social care services.

RQIA was established in 2005. It is the main scrutiny body in Northern Ireland’s care system and provides independent assurance about the quality of health and social care services.

In England, the regulatory architecture is more fragmented and there are areas of overlap. There are three main healthcare provider regulatory agencies, the CQC, TDA and Monitor. The CQC was formed as a single integrated regulatory agency in 2009 from a merger of predecessor organisations. CQC’s purpose is to ensure health and social care services provide people with high-quality care and to encourage improvement (Care Quality Commission, 2013).

The English National Health Service has been pursuing a policy to develop Foundation Trusts (Walshe, 2003a), which have more independence from the Department of Health provided a number of criteria is met. Monitor is the sector regulator of Foundation Trusts in England, a non-departmental public body of the Department of Health, established in 2004, The TDA is a special health authority of the Department of Health set up following the Health and Social Care Act in 2012. It provides the oversight, scrutiny and performance management of non-Foundation Trusts on behalf of the Department of Health and develops them into Foundation Trusts. The TDA does not have formal regulatory powers.

All the regulatory agencies were established from 2004 onwards with the most recent being the TDA in 2012. All, excepting the TDA, have seen growth in their scope since establishment. This has often followed emerging quality failures, for example in Lanarkshire, Scotland, (Healthcare Improvement Scotland, 2013b). All four countries have held inquiries into cases of poor care, which have affected scope, responsibilities and the regulatory model used (Table II).

It is not clear how the agencies choose the processes they use to discharge their regulatory responsibilities but often this seems to be in reaction to national and political context rather than through a deliberative process. This suggests that the regulatory agencies are path-dependent in how they deliver their regulatory aims of improvement, assurance and accountability, reacting to the external environment rather than making an explicit choice of regulatory model. The paper analyses the specific goals and regulatory models of each agency. Table III analyses the documents and interviews to compare regulatory goals and models, and shows that there are three “hybrid” regulatory agencies. Agencies demonstrate aspects of several regulatory models making model categorisation challenging to complete. The term “hybrid” is used to illustrate an emergent responsive regulatory approach whereby regulatory agencies are primarily using enforcement methods that comprise of improvement support through direct action that is tailored contingent on organisational circumstances and performance. The remaining agencies described methods that remained invariant, regardless of organisational circumstances and because the enforcement methods used did not include the provision of improvement support.

### Tensions within hybrid models

The analysis highlights a tension caused through the combination of assurance, accountability and improvement goals:

We’re part of the architecture that can make organisations simply focus on the problem of today, […] [whereas] organisations need to find that balance between addressing today [and] tomorrow

(Interviewee F, TDA).

[…] it’s quite clear that we’re there to scrutinise and to regulate, but we’re also there to try to help improvement […] it isn’t always easy to fit the two together

(Interviewee H, CQC).

[NHS] Boards are saying actually don’t confuse us. You can’t come in with an inspection hat on and then an improvement one

(Interviewee C, HIS).

This paper identifies three themes from this tension between compliance and improvement support within emerging hybrid models – regulatory role, resources and relationships – and we now discuss each in turn.

### Regulatory roles

Interviewees and documents describe a tension between the roles of assuring the public of safe, quality care and improving care:

Quality care cannot be achieved by inspection and regulation alone. The main responsibility for delivering quality care lies with [those that provide], arrange and fund local services

(Care Quality Commission, 2013).

The Berwick report (2013) highlights the vital role that ‘intelligent inspection’ plays. However, this cannot stand alone and must be combined within a system of improvement

(Healthcare Improvement Scotland, 2014).

We’re very clear what our role is when we go in and our role is not to run the trust or run a piece of work

(Interviewee A, TDA).

They’re their own problems, because if we solve it for them, then they haven’t worked it through, and I couldn’t solve it

(Interviewee A, RQIA).

Some agencies are concerned that delivering improvement activity compromises their “role” to conduct objective detection. Interviewees also raise concerns regarding accountability should the improvement support not lead to the expected outcomes:

[…] there is a danger of conflict, that we mark our own homework […] a hospital [could] say, but you’ve been working with us on this so the failure is also partly yours

(Interviewee A, Monitor).

When trusts aren’t performing, there is a lot of pressure in the system, to say […] to almost indicate that it’s wilful. It’s almost as if they’re failing for reasons which they should be able to stop

(Interviewee C, TDA).

We don’t make standards because it would be an uncomfortable place to be, to be the regulator and review against your own standards

(Interviewee E, RQIA).

### Resources

The choice of regulatory approach has important ramifications for planning and execution, as it affects the type of resources (e.g. information technology vs clinical skills) and experienced staff that are needed by the regulatory agency, and influences the financial resources available for other regulatory tasks. Compliance models, for example, need more inspectors whereas hybrid models need more improvement facilitators. This makes the choice of regulatory model more path-dependent and slow to change. Analysis suggests that that relatively few employees may have improvement skills or experience within regulatory agencies. Shortages need addressing through development, recruitment and investment:

We had no resources to take it forward

(Interviewee B, HIS).

We’ve got quite a big, sort of, issue about needing to invest in our staff […] you can’t just outsource […] we just don’t have the time and need some supplemental space to be able to really engage with [improvement]. So, it is quite a big challenge for us

(Interviewee A, CQC).

There [is] a challenge to find people of those skills

(Interviewee B, HIW).

It is clear from the interviews and documents that some regulatory staff resist the development of hybrid models. This may be due to the lengthy period and costs of developing skills, or to disagreements regarding the regulatory aims and concerns regarding local accountability:

[I wonder] how knowledgeable the inspectors are around improvement methodology because you can’t judge it unless you know what you’re looking for […] I think the inspectors lack the improvement methodology understanding […] we don’t have the special advisors either

(Interviewee C, CQC).

We haven’t got anything like the number of people working within Monitor that have the [improvement] experience they’d need […] some people would say, this isn’t a job for a regulator

(Interviewee F, Monitor).

RQIA has limited capacity […] to encourage service providers to continuously improve

(Regulation and Quality Improvement Authority, 2015a, b).

Regulatory agencies report pressures linked to resources and describe a trade-off required between detection and enforcement activities and the resources available:

[…] we would have to think carefully about whether our time’s better spent doing [improvement work] or another inspection somewhere else

(Interviewee B, HIW).

[…] with regulation, you have to prioritise, if we were regulating everybody it wouldn’t have any impact and [we] wouldn’t have enough resources

(Interviewee B, Monitor).

### Relationships

Interviewees comment on their need to maintain effective working relationships with organisations and to establish trust and openness to assure the public that their assessments of care quality are fair, trustworthy and accurate. However, interviewees acknowledge the risks of negative reporting, noting that detection and enforcement together with tough media and political scrutiny can develop destabilising effect on organisations and associated relationships:

[…] if you establish good ongoing relationships outside the inspection regime then it’s less about you coming in and more about the team that the hospital knows

(Interviewee C, CQC).

You’re still having that professional distance as a regulator but you get to know the chief exec […] and they get to know you

(Interviewee F, HIW).

[…] the approach of some providers might be […] they’re a regulator so I don’t want to go near them whereas some of our best relationships with trusts are […] coming to us very early for advice

(Interviewee B, Monitor).

However, analysis of documents indicates that agencies believe that enforcement action, both punitive and supportive, must be transparent to prevent against regulatory capture to maintain public trust in “independent and objective” regulators:

HIW will report clearly, openly and publicly on the work that we undertake in order that citizens are able to access independent and objective information on the quality, safety and effectiveness of healthcare in Wales

(Healthcare Inspectorate Wales, 2014a; Healthcare Inspectorate Wales, 2014b).

By publicly reporting our ﬁndings, we provide assurance to the public that standards are being met, or that action is being taken where improvements are needed

(Healthcare Improvement Scotland, 2013a).

These two contrasting perspectives, of confidentiality and openness, can be difficult to reconcile:

There is an inherent tension with that confidential, closed-doors enquiry support with the requirements for us as a body about public accountability and transparency

(Interviewee G, HIS).

Finally, external stakeholders such as the media may use information differently, hindering relationship development, mutual trust and care improvement in some circumstances. Those providing care may be concerned that information disclosure may deter honest discussion of problems due to these stakeholders (Berwick *et al.*, 2003).

## 5. Discussion and implications

This paper describes how regulatory agencies in the four countries of the UK have different organisational remits, scope, approaches and methods of delivering their mission. The analysis suggests that effective regulatory oversight relates to the ability of regulatory agencies to balance the requirements to assure and improve care. Hybrid regulatory models are emerging in response, such as the approach taken by HIS, Monitor and the TDA. Hybrid regulatory models have to balance multiple identities which can create conflicts linked to roles and identities, resources and relationships. But hybrid organisations are sometimes described as unstable and may fracture and revert to dominant roles and identities under sustained pressure (Denis *et al.*, 2015). Hybrid regulatory agencies need to find ways to manage the identified tensions to sustain the balance of their requirements to assure and improve care.

Hybrid regulatory models require a range of resources in order to deliver improvement support as well as to provide assurance. Hybrid regulatory models require the regulatory agency to be able to differentiate between organisations and tailor regulatory interventions accordingly. For example, do all organisations require improvement support or only those who have poor performance or high risk levels, or is proactive improvement support offered to all organisations regardless of performance to prevent future poor performance? How should this be prioritised? It might be argued that regulatory agencies seeking to use hybrid regulatory models need to do more to articulate their underlying improvement model (Davidoff *et al.*, 2015).

There remains a risk that high levels of intervention and support for improvement could jeopardise the trustworthiness of the regulator as an independent assessor, strain relationships and blur roles and accountabilities. Moreover, if the main motivation within organisations for improvement derives from external regulation, organisations may exert less effort into implementation (Piening, 2011). This could inhibit healthcare organisations from investing and developing long-term improvement capability of their own, leading to a dependence on external improvement support from the regulatory agency and increasing their resource requirements.

Instead of providing high levels of on-going intervention and support for improvement, healthcare regulatory agencies could strengthen their approaches to assure and improve care by focusing on the development of improvement capability as well as seeking to ensure compliance with standards and performance within regulated organisations. This could help to ensure that regulatory agencies are supporting the development of more proactive approaches to the improvement of quality without directly doing improvement work for or to organisations, allowing regulatory agencies to benefit from the advantages of hybridity whilst limiting some of the risks outlined above.

## 6. Conclusion

Effective healthcare regulation requires recognition of the inherent tensions between the regulatory aims of improvement, accountability and assurance. Hybrid regulatory models are emerging within UK regulatory agencies to assure and improve care, and these use direct improvement support for organisations to supplement other regulatory interventions. This paper identifies that the development of hybrid models is complex and emergent. There are three key areas of challenge linked to roles, resources and relationships when developing and sustaining hybrid models. This paper contributes to research by presenting findings furthering the understanding and emergence of hybrid models in healthcare regulation.

## Figures and Tables

**Table I:** Agency comparison

Country and population	Name	Staff (WTE)	Expenditure
Scotland: 5.3M	Healthcare Improvement Scotland (HIS)	329	£20M (14/15)
Wales: 3M	Healthcare Inspectorate Wales (HIW)	59	£3M (14/15)
Northern Ireland: 1.8M	Regulatory & Quality Improvement Authority (RQIA)	152	£7.6M (13/14)
England: 53M	Care Quality Commission (CQC)	2,681	£240M (14/15)
England: ~149 Foundation Trusts (FTs)	Monitor	532	£72.3M (14/15)
England: ~90 Non-Foundation Trusts (non-FTs)	Trust Development Authority (TDA)	315	£65M (14/15)

**Table II:** Impact of responses to quality issues on regulatory agencies

Agency	Issue	Response	Impact
HIS	High Mortality Rates at NHS Lanarkshire	Review of NHS Lanarkshire (Healthcare Improvement Scotland, 2013b)	Leading to development of new scrutiny approach – “Quality of Care Reviews”
HIW	Care concerns at Abertawe Bro Morgannwg University (ABMU) Health Board and wider concerns about effectiveness of HIW	Trusted to Care Independent Review (Andrews and Butler, 2014); HIW Review (Marks, 2014)	Independent review of concerns at ABMU and the Welsh Health and Social Care Committee review of HIW in 2013. Followed by a formal review of HIW (Marks, 2014)
RQIA	Incidents at Belfast Health and Social Care Trust and Northern Care Health and Social Care Trust	Instigated reviews by RQIA of the Trusts. The Minister in parallel initiated a review of the Northern Irish health and social care system (Donaldson *et al.*, 2014)	The review of the health and social care system found that RQIA had little visibility and the healthcare system needed to strengthen its approach to improving quality
CQC	High Mortality Rates and patient neglect at Mid Staffordshire NHS Foundation Trust, similar failings in care at Winterbourne View and Morecambe Bay FT	The Mid Staffordshire Enquiry (Francis, 2013) Morecambe Bay Enquiry (Kirkup, 2015) Winterbourne View (Department of Health, 2012)	Development of new inspection approach based on the NHS England reviews of high mortality trusts conducted in response to the Francis Enquiry
Monitor	As CQC	As CQC	Change in role following 2012 Health and Social Care Act
TDA	As CQC	TDA did not exist during the time of these issues; however, the impact of them influenced the design of the organisation	Established following 2012 Health and Social Care Act

**Table III:** Agency goals and models

Agency	Documentary data	Interview data	Agency model
HIS	“We are the national healthcare improvement organisation for Scotland, established to advance improvement in healthcare” (Healthcare Improvement Scotland, 2014)	“[…] a blend of approaches: so we have the scrutiny, assurance, we have the clinical expertise […] independent fair and objective assessment […] [and] […] support improvement efforts” (Interviewee G, HIS) “[we] […] help providers in Scotland to improve their improvement capability (Interviewee A, HIS)	Hybrid
HIW	“Our purpose is to provide independent and objective assurance on the quality, safety and effectiveness of healthcare services, making recommendations to healthcare organisations to promote improvements” (Healthcare Inspectorate Wales, 2014)	“we go out and inspect and we find […] an organisation is meeting the standards […] then we wouldn’t seek improvement […] beyond that (Interviewee B, HIW) “we are not an improvement agency, but we should be operating in a way which supports improvement” (Interviewee D, HIW)	Compliance
RQIA	“The most important priority for RQIA is to make sure that our inspection systems and processes convey clearly to the public how well a service is performing in respect of the […] minimum standards” (Regulation and Quality Improvement Authority, 2015a, b)	“We provide assurance […] about the quality of services” (Interviewee D, RQIA) “our primary role is to question them, to challenge them early, and then they can then start making […] improvements” (Interviewee A, RQIA)	Compliance
CQC	“We make sure health and social care services provide people with safe, effective, compassionate, high-quality care and we encourage care services to improve” (Care Quality Commission, 2013)	“We monitor, we inspect and we regulate and make sure that these services meet the fundamental standards” (Interviewee CQC D) “it’s very clear in the CQC that we’re not improvement facilitators, we’re regulators” (Interviewee C, CQC)	Compliance
Monitor	“[We set] a required standard that all NHS providers must meet […] [We] control the risk that foundation trusts, once authorised, fall back below the required standard. If they do, we take remedial action […] We will focus in particular on the capabilities that drive long-term performance” (Monitor, 2014)	“where trusts fail to deliver certain minimum standards […] [we] work with those trusts to ensure that they improve their position and restore themselves to […] that minimum standard” (Interviewee A, Monitor) “[Our] mandate is basically to improve the capability of FTs” (Interviewee G, Monitor)	Hybrid
TDA	“The TDA oversees NHS trusts and holds them to account […] while providing them with support to improve” (Trust Development Authority, 2014)	“[Trusts] know that they are being held to account for their performance but they also know that they will get support and help and development rather than just being criticised” (Interviewee G, TDA) “[Our role is] supporting oversight of our Trusts, […] [and] that have asked for some support because they feel that they need to make some improvements” (Interviewee E, TDA)	Hybrid

## Note

1. Since this research was completed the TDA and Monitor have been merged with the operational name of NHS Improvement, though the underlying legislation which created them has not been revised, so they still exist statutorily as two separate organisations.
